# UPLC-HRMS Analysis Revealed the Differential Accumulation of Antioxidant and Anti-Aging Lignans and Neolignans in *In Vitro* Cultures of *Linum usitatissimum* L

**DOI:** 10.3389/fpls.2020.508658

**Published:** 2020-09-23

**Authors:** Shankhamala Bose, Thibaut Munsch, Arnaud Lanoue, Laurine Garros, Duangjai Tungmunnithum, Souhila Messaili, Emilie Destandau, Kévin Billet, Benoit St-Pierre, Marc Clastre, Bilal Haider Abbasi, Christophe Hano, Nathalie Giglioli-Guivarc’h

**Affiliations:** ^1^ EA2106 Biomolécules et Biotechnologies Végétales, Université de Tours, Tours, France; ^2^ UMR7311, Institut de Chimie Organique et Analytique, Université d’Orléans, CNRS, Orléans, France; ^3^ USC1328 Laboratoire de Biologie des Ligneux et des Grandes Cultures, Université d’Orléans, INRA, Orléans, France; ^4^ Department of Pharmaceutical Botany, Mahidol University, Bangkok, Thailand; ^5^ Department of Biotechnology, Quaid-i-Azam University, Islamabad, Pakistan

**Keywords:** lignans, neolignans, callus, cell culture, antioxidant activity, anti-aging activity

## Abstract

Over the last few decades, methods relating to plant tissue culture have become prevalent within the cosmetic industry. Forecasts predict the cosmetic industry to grow to an annual turnover of around a few hundred billion US dollars. Here we focused on *Linum usitatissimum* L., a plant that is well-known for its potent cosmetic properties. Following the a) establishment of cell cultures from three distinct initial explant origins (root, hypocotyl, and cotyledon) and b) selection of optimal hormonal concentrations, two *in vitro* systems (callus *vs* cell suspensions) were subjected to different light conditions. Phytochemical analysis by UPLC-HRMS not only confirmed high (neo)lignan accumulation capacity of this species with high concentrations of seven newly described (neo)lignans. Evaluation over 30 days revealed strong variations between the two different *in vitro* systems cultivated under light or dark, in terms of their growth kinetics and phytochemical composition. Additionally, antioxidant (*i.e.* four different *in vitro* assays based on hydrogen-atom transfer or electron transfer mechanism) and anti-aging (*i.e.* four *in vitro* inhibition potential of the skin remodeling enzymes: elastase, hyaluronidase, collagenase and tyrosinase) properties were evaluated for the two different *in vitro* systems cultivated under light or dark. A prominent hydrogen-atom transfer antioxidant mechanism was illustrated by the DPPH and ABTS assays. Potent tyrosinase and elastase inhibitory activities were also observed, which was strongly influenced by the *in vitro* system and light conditions. Statistical treatments of the data showed relationship of some (neo)lignans with these biological activities. These results confirmed the accumulation of flax (neo)lignans in different *in vitro* systems that were subjected to distinct light conditions. Furthermore, we showed the importance of optimizing these parameters for specific applications within the cosmetic industry.

## Introduction

Records on the cosmetic usage of natural plant products date back to ancient times as described in famous texts, including 1) Ayurvedic books on traditional Indian medicine (3,000–2,000 BC), 2) Chinese pharmacopoeia, 3) “The Divine Farmer’s Herb-Root Classic”, by Shen Nong (3,494 BC), and 4) the ancient Greek book “De Materia Medica”, written by Pedanius Dioscorids (Ota and Yokoyama, 2010). However, regardless of a vast and well-established ethnobotanical knowledge base, very few plants have been thoroughly investigated in modern times for their potential use within the cosmetic industry (Fongnzossie et al., 2017).

Within this modern era of technology and globalization, it is quite surprising to find a shift in consumer preference from chemical based synthetic products to more natural or “green” products. This could perhaps be explained by the increasing concerns of side effects that are associated with chemical products (Hazra and Panda, 2013). However, due to a recent increase in the global demand for active plant ingredients within the medical and cosmetic industries, medicinal plant species are now experiencing severe mass exploitation which may ultimately lead to their extinction. *In vitro* plant tissue culture technology can aid in supplying the growing global demand for active biomolecules, without over exploitation of plant biomes (Barbulova et al., 2014).

Moreover, the use of plant cell cultures instead of cultivated plants for active biomolecule production may help to overcome limitations of inconsistent quality due to seasonal changes, cultivation methods and geographic variations. Batch to batch inconsistencies can also be avoided by creating an environment that is free of pathogens and contamination (Barbulova et al., 2014). Furthermore, due to the controlled conditions associated with this technology, it can also be successfully applied to increase the amount of active biomolecule production by using biotransformation techniques and/or elicitation of stress (biotic and abiotic) conditions.

*Linum usitatissimum* L., often referred to as flax, is a commercially important plant, belonging to the Linaceae family. Its literature dates back to 5,000 BC when it was primarily cultivated for fiber and oil in Western Europe, Mediterranean region, North Africa and South-West Asia (Oomah, 2001; Zohary et al., 2012). Recent studies of *L. usitatisimum* have elucidated several useful properties of the plant, including anticancer (Shim et al., 2014), anti-diarrhea (Palla et al., 2015) anti-microbial (Bakht et al., 2011), anti-inflammatory (Oomah, 2001), antioxidant and prevention against cardiovascular diseases (Zanwar et al., 2011). Part of these health benefits has been associated with the presence of (neo)lignans in flax. Following their consumption, plant lignans are converted to enterolignans (enterolactone and enterodiol) by intestinal microbes in the gut which have been reported to reduce the occurrence of different cancers (Lainé et al., 2009; Zanwar et al., 2011).

There are many reports describing *in vitro* tissue culture systems used for propagating *L. usitatisimum* from hypocotyl (Cunha and Ferreira, 1999; Dedičová et al., 2000; Salaj et al., 2005) and anther (Nichterlein et al., 1991; Rutkowska-Krause et al., 2003) explants. Additionally, callus (Anjum et al., 2017a; Zahir et al., 2018) and cell suspension cultures (Attoumbré et al., 2006a; Attoumbré et al., 2006b; Hano et al., 2006; Beejmohun et al., 2007; Hano et al., 2008; Corbin et al., 2013a; Corbin et al., 2013b; Gabr et al., 2016; Anjum et al., 2017b; Nadeem et al., 2018; Nadeem et al., 2019; Ahmad et al., 2019; Markulin et al., 2019) producing higher amounts of industrially important lignans and neolignans have also been described.

Flax is considered to be a potential cosmetic ingredient all over the world, including China (China Food & Drug Administration, 2015). It is therefore surprising that this multifunction and economically important crop has hardly been exploited within the cosmetic industry. Thus, the main objective of this study was to establish cell lines (solid and liquid) of *L. usitatissimum*, producing valuable specialized metabolites of great importance for cosmetics.

## Materials and Methods

### Chemicals and Reagents

The extraction solvents used in this experiment were of analytical grade, supplied by Thermo Scientific (Courtaboeuf, France), while all other standards and reagents were purchased from Sigma-Aldrich (Saint-Quentin Fallavier, France).

### Plant Material and Establishment of Callus

The selection of explants (hypocotyls, cotyledons, and roots) and callus formation was achieved following the protocol described by Hano et al. (2006), with slight modifications. Briefly, hypocotyl explants were chosen, and the best growing callus was found to be in Murashige and Skoog (1962) media containing 2 mg.L^−1^ BAP and 0.5 mg.L^−1^ NAA. Cultures were maintained under two illumination conditions: 1) one with 12 h light with 25 µE m^−2^ s^−1^ light intensity/12 h dark and 2) the other in total darkness (24 h per day). Light intensity was measured by using Luxmeter under the light source. Illumination was ensured by dark red/white LED (18 W, Green Power TLED DR/W, Philips). The growth room temperature was maintained at 24°C for both conditions. The callus was subcultured after every 30 days for both conditions.

### Establishment of Suspension Culture

For initiation of cell suspension culture, approximately 1 g of fresh weight (FW) callus was added to a 125 ml Erlenmeyer flask containing 25 ml of MS media fortified with 2 mg.L^−1^ benzylaminopurine (BAP) and 0.5 mg.L^−1^ 1-Naphthaleneacetic acid (NAA). The suspension cultures were kept on a gyratory shaker at 120 rpm, at 24°C. Then suspension cultures were maintained either in 12 h light/12 h dark or 24 h darkness and subcultured every 14 days.

### Study of Growth Kinetics

For analysis of growth kinetics (callus and cell suspension culture), 10 sampling points were studied over a period of 30 days with 3 interval days.

For the determination of the FW of the suspension culture, the cells were harvested by filtration using a 0.45 μm stainless steel sieve, then washed twice with distilled water to remove any trace of medium. In order to remove the excess adhering water, the cells are manually pressed between two filter papers several times until no more traces of liquid are visible on the papers. The cells are then weighed. For dry weight (DW) estimation, cells were frozen and lyophylized 48 h (lyophilizator CHRIST Alpha 1-5) and then weighed.

### Plant Extract Preparation

Dried cells were ground to a fine powder with a mortar and pestle. Fifty milligrams of the powder was extracted in 1 ml ethanol/water solution (75%, v/v) in a sonication bath for 1 h. The extracts were then centrifuged at 18,000 g for 10 min. Supernatant (500 µl) was collected and stored at −20°C for performing bioassays and metabolic profiling.

### UPLC-MS Analyses

For detection of phenolics, lignans, and neolignans, UPLC-MS analyses were performed according to Billet et al. (2018). Briefly, the analysis was performed on an ACQUITY™ Ultra Performance Liquid Chromatography system coupled to a photo diode array detector (PDA) and a Xevo TQD mass spectrometer (Waters, Milford, MA). The Xevo TQD was controlled by MassLynx 4.1 software (Waters, Milford, MA) and equipped with an electrospray ionization (ESI) source. Sample separation was accomplished by Waters Acquity HSST3 C18 column (150 × 2.1 mm, 1.8 μm) with a flow rate of 0.4 ml.min^−1^ at 55°C. The injection volume was 5 μl. The mobile phase consisted of solvent A (0.1% formic acid (FA) in water) and solvent B (0.1% formic acid in acetonitrile). Chromatographic separation was accomplished using a 19-min linear gradient from 5 to 60% mobile phase B. Mass spectrometry (MS) detection was performed in both positive and negative ionization modes, the source temperature being 120°C and the desolvation temperature 350°C. The capillary voltage was 3,000 V, and sample cone voltages were 30 and 50 V in full scan mode. The cone and desolvation gas flow rates were 60 and 800 L.h^−1^ respectively. Analytes were annotated according to their retention time, UV, and mass spectra by comparison with pure commercial standards and data from the literature (**Table S1**). Integration of the peaks was done using TargetLynx software. Targeted data collection was carried in selected ion monitoring (SIM) mode for (1) *erythro*-guaiacylglycerol-*β*-coniferyl alcohol ether glucoside ([M+H-2H_2_O]^+^; *m*/*z* 521; RT = 5.66 min), (2) *threo*-guaiacylglycerol-*β*-coniferyl alcohol ether glucoside ([M+H-2H_2_O]^+^; *m*/*z* 521; RT = 5.79 min), (3) *p*-coumaric ([M-H]^−^; *m*/*z* 163; RT = 6.49 min), (4) dehydrodiconiferyl alcohol-4-*β*-D-glucoside isomer 1([M+H-H_2_O]^+^; *m/z* 503; RT = 7.3 min), (5) *erythro*-guaiacylglycerol-*β*-coniferyl alcohol ether ([M+H-2H_2_O]^+^; *m*/*z* 341; RT = 7.69 min), (6) *threo*-guaiacylglycerol-*β*-coniferyl alcohol ether ([M+H-2H_2_O]^+^; *m*/*z* 341; RT = 7.85 min), (7) dehydrodiconiferyl alcohol-4-*β*-D-glucoside isomer 2 ([M+H-H_2_O]^+^; *m/z* 503; RT = 8.3 min), (8) secoisolariciresinol ([M+H-2H_2_O]^+^; *m/z* 327; RT = 9.42 min), (9) lariciresinol ([2M+H]^+^; *m*/*z* 721; RT = 9.77 min), (10) epipinoresinol ([M+H-H_2_O]^+^ *m*/*z* 341; RT = 9.9 min), (11) pinoresinol ([M+H-H2O]+; *m/z* 165; RT = 10.48 min), (12) pluviatolide ([M+H]^+^; *m*/*z* 357; RT = 11.36 min), (13) guaiacylglycerol-*β*-coniferyl aldehyde ether hexoside ([M+H-2H_2_O]^+^; *m*/*z* 519; RT = 11.74 min), (14) phillygenin ([M-H]^−^; *m*/*z* 371; RT = 13.23 min). Peak integration was performed using the ApexTrack algorithm with a mass window of 0.1 Da and relative retention time window of 1 min followed by Savitzky–Golay smoothing (iteration = 1 and width = 1). The resulting pairs of m/z values and retention times were also manually examined.

As there are limited reference mass spectra available for lignan and neolignan identification, high-resolution mass spectrometry was further employed for confirmation of UPLC-DAD-MS identification. Chromatographic analyses were performed using an Ultimate 3000 RSLC system equipped with a binary pump, an autosampler and a thermostated column compartment (Dionex, Germering, Germany). Analytes were separated on a Luna omega C18 column (150 × 2.1 mm; 1.6 µm, Phenomenex) at 40°C. The mobile phase at a flow rate of 500 µl.min^−1^ was composed of solvent A (0.1% formic acid in water) and solvent B (0.08% formic acid in acetonitrile); the gradient program was as follows: 97% A and 3% B from 0 to 3 min, 55% A and 45% B at 12 min, 10% A and 90% B from 14 to 15 min, 97% A and 3% B at 15.5 min, then the column was re-equilibrated under initial conditions during 3 min. The injection volume was 2 µl. MS experiments were performed on a maXis UHR-Q-TOF mass spectrometer (Bruker, Bremen, Germany) in positive and negative electrospray ionization (ESI) modes. Capillary voltage was set at 4.5 kV in positive mode and 4.0 kV in negative mode. The flow rates of nebulizing and drying gas (nitrogen) were respectively set at 2 bars and 9 L.min^−1^, and drying gas was heated at 200°C. The analysis was made with an acquisition frequency of 0.6 Hz for MS and MS/MS; the mass scan range was set from *m/z* 50 to 1,550. MS/MS experiments were carried out using data dependent acquisition (DDA) mode. Two collision energies were applied according to *m/z*, and the spectra were averaged to obtain MS/MS spectra from 20 and 45 eV at *m/z* 140 to 35 and 78 eV at *m/z* 1,000. Data were processed using DataAnalysis 4.4. The molecular formula was calculated using the following parameters: elemental composition ^12^C, ^1^H, ^16^O, ^14^N_0-5_ and mass accuracy ≤2 ppm. The HRMS data for the 14 identified metabolites are presented in **Table 1**.

**Table 1:** UHPLC-HR-ESI-MS data of *Linum usitatissimum* cell suspension extracts.

Peak	RT (min)	Compound class	CompoundAssignement	Molecular formula	m/z measured	m/z calculated	Error [ppm]
1	5.68	neolignan	*erythro*-guaiacylglycerol-*β*-coniferyl alcohol ether glucoside	C26H33O11	521.202052 [M+H-H2O]^+^	521.201738	−0.6
2	5.80	neolignan	*threo*-guaiacylglycerol-*β*-coniferyl alcohol ether glucoside	C26H33O11	521.201547 [M+H-H2O]^+^	521.201738	0.4
3	4.26	phenolic acid	*p*-coumaric acid	C9H9O3	165.054376 [M+H]^+^	165.054621	1.5
4	7.19	neolignan	dehydrodiconiferyl alcohol-4-*β*-D-glucoside isomer1	C26H31O10	503.1909 [M+H-H2O]^+^	503.191174	1.6
5	7.35	neolignan	*erythro*-guaiacylglycerol-*β*-coniferyl alcohol ether	C20H24NaO7	399.14164 [M+Na]^+^	399.14142	−0.5
6	7.52	neolignan	*threo*-guaiacylglycerol-*β*-coniferyl alcohol ether	C20H24NaO7	399.14150 [M+Na]^+^	399.14142	−0.2
7	7.80	neolignan	dehydrodiconiferyl alcohol-4-*β*-D-glucoside isomer2	C26H33O11	521.201475 [M+H]^+^	521.201738	1.6
8	6.78	dibenzylbutane	secoisolariciresinol	C20H27O6	363.180449 [M+H]^+^	363.180215	−0.6
9	9.31	furan	lariciresinol	C20H24NaO6	383.146804 [M+Na]^+^	383.146509	−0.8
10	9.37	furofuran	epipinoresinol	C20H23O6 C20H22NaO6	359.148467 [M+H]^+^ 381.131 [M+Na]^+^	359.148915 381.130859	1.2 −0.4
11	9.74	furofuran	pinoresinol	C20H22NaO6	381.131389 [M+Na]^+^	381.130859	−1.4
12	10.68	dibenzylbutyrolactone	pluviatolide	C20H21O6	357.133699 [M+H]^+^	357.133265	−1.2
13	10.93	neolignan	guaiacylglycerol-*β*- coniferyl aldehyde ether hexoside	C30H33O9	537.211577 [M+H]^+^	537.211909	0.6
14	12.09	furofuran	Phillygenin	C21H24NaO6	395.146532 [M+Na]^+^	395.146509	−0.1

All the 14 metabolites identified were followed during growth kinetic in callus and cell suspensions, and the relative abundance of each metabolite is estimated according Arbitrary Unit (AU) by mg of DW.

### Antioxidant Activity

#### DPPH Radical Scavenging Assay

To determine the antioxidant activity in the cell culture extracts, the 2,2-Diphenyl-1-picrylhydrazyl (DPPH) antioxidant free radical scavenging assay was performed according to the method described by Lee et al. (1998). Briefly, 20 µl of cell extract was mixed with 180 µl of DPPH reagent and kept for 30 min in the dark at room temperature, after which the absorbance was noted using a microplate reader at 517 nm. Trolox C was used as positive control. The assay was performed in triplicate and results expressed in μM of Trolox C Equivalent Antioxidant Capacity (TEAC) using a 6-point calibration curve (R^2^ = 0.9994).

#### ABTS Radical Scavenging Assay

This assay, also known as Trolox equivalent antioxidant capacity assay, was performed with the 2,2′-azinobis-(3-ethylbenzothiazoline-6-sulfonate) (ABTS) radical as described by Tagliazucchi et al. (2010) with slight modifications. Briefly, equal volumes of 7 mM ABTS solution were added to 2.45 mM potassium persulphate solution and incubated in the dark for 16 h at room temperature. Next, the absorbance was recorded at 734 nm and adjusted to 0.7, after which the extracts were added. The reaction was then kept in the dark for 15 min at 25°C, and the absorbance was measured again at 734 nm by the use of BioTek ELX800 absorbance microplate reader (BioTek Instruments, Colmar, France). Trolox C was used as positive control. The assay was performed in triplicate and results expressed in μM of Trolox C Equivalent Antioxidant Capacity (TEAC) using a 6-point calibration curve (R^2^ = 0.9977).

#### FRAP Assay

Ferric reducing antioxidant power assay (FRAP) was carried out according to the protocol described by Benzie and Strain (1996) with small modifications. Briefly, 10 µl of sample plant extract was added to 190 µl of FRAP solution, which was composed of 20 mM FeCl_3_ 10 mM TPTZ, 6H_2_O, along with 300 mM acetate buffer (pH 3.6) in 1:1:10 (v/v/v) ratio. The reaction mix was incubated for 15 min at 25°C. The absorbance was then measured at 630 nm using a BioTek ELX800 absorbance microplate reader (BioTek Instruments, Colmar, France). Trolox C was used as positive control. The assay was performed in triplicate and results expressed in μM of Trolox C Equivalent Antioxidant Capacity (TEAC) using a 6-point calibration curve (R^2^ = 0.9941).

#### CUPRAC Assay

A modified method of Apak et al. (2004) was used to determine the cupric ion reducing antioxidant capacity (CUPRAC) of the samples. Briefly, 10 µl of sample plant extract was mixed with 190 µl of CUPRAC solution, containing 10 mM Cu(II), 7.5 mM neocuproine, and 1 M acetate buffer (pH 7.0) in a 1:1:1 (v/v/v) ratio. The mixture was then incubated for 15 min at 25°C and the absorbance recorded at 450 nm using the BioTek ELX800 absorbance microplate reader (BioTek Instruments, Colmar, France). Trolox C was used as positive control. The assay was performed in triplicate and results expressed in μM of Trolox C Equivalent Antioxidant Capacity (TEAC) using a 6-points calibration curve (R^2^ = 0.9997).

### Anti-Aging Activity

#### Collagenase Assay

The collagenase assay was performed according to Wittenauer et al. (2015). Collagenase from *Clostridium histolyticum* (Sigma Aldrich) was used with the substrate N-[3-(2-furyl)acryloyl]-Leu-Gly-Pro-Ala (FALGPA; Sigma Aldrich) and the decrease in absorbance of FALGPA was monitored at 335 nm over a period of 20 min, using a BioTek ELX800 absorbance microplate reader (BioTek Instruments, Colmar, France). All the reactions were performed in triplicate, and the anti-collagenase activity was detected as a percentage of inhibition relative to the control (by adding the same volume of extraction solvent) for each extract. 1,10-Phenantroline (100 µM) was used as the specific inhibitor of collagenase leading to an inhibition of 33.6 ± 2.2%.

#### Elastase Assay

For this assay, porcine pancreatic elastase (Sigma Aldrich) was used according to the protocol described by Wittenauer et al. (2015). Here, N-Succ-Ala-Ala-Ala-*p*-nitroanilide (AAAVPN; Sigma Aldrich) was used as a substrate, and the release of *p*-nitroaniline was measured at 410 nm using an absorbance microplate reader (BioTek ELX800; BioTek Instruments). All the experiments were performed in triplicate, and the anti-elastase activity was expressed as a percentage of inhibition relative to the control which consisted of the same volume of extraction solvent. Oleanolic acid (10 µM) was used as the specific inhibitor of elastase leading to an inhibition of 47.8 ± 1.4%.

#### Hyaluronidase Assay

The assay for hyaluronidase inhibitory action was carried out as described by Kolakul and Sripanidkulchai (2017). For the reaction, 1.5 units of hyaluronidase (Sigma Aldrich) was added to the substrate *i.e.* 0.03% (w/v) hyaluronic acid solution, after which, acid albumin solution [0.1% (w/v) BSA] was used to precipitate undigested form of hyaluronic acid. The absorbance was recorded at 600 nm using an absorbance microplate reader (BioTek ELX800; BioTek Instruments, Colmar, France). All the experiments were performed in triplicate, and the hyaluronidase inhibitory action was expressed as a percentage of inhibition relative to the control which consisted of the same volume of extraction solvent. Oleanolic acid (10 µM) was used as the specific inhibitor of hyaluronidase leading to an inhibition of 33.5 ± 2.8%.

#### Tyrosinase Assay

Tyrosinase inhibitory assay was carried out as described by Chai et al. (2018). Briefly, the diphenolase substrate l-DOPA (5 mM; Sigma Aldrich) was mixed in sodium phosphate buffer (50 mM, pH 6.8) with 10 µl of *L. usitatissimum* extract after which, 0.2 mg.ml^−1^ of mushroom tyrosinase solution (Sigma Aldrich) was added to the reaction mixture to make a final volume of 200 µl. A control experiment was performed in parallel using an equal amount of extraction solvent. The absorbance of the reaction was measured using an absorbance microplate reader (BioTek ELX800; BioTek Instruments) at 475 nm. All the experiments were performed in triplicate, and the hyaluronidase inhibitory action was expressed as a percentage of inhibition relative to the control for each extract. Kojic acid (10 µM) was used as the specific inhibitor of tyrosinase leading to an inhibition of 51.2 ± 0.9%.

#### Statistical Analysis

Visualization of the data and data analysis were carried out with MeV 4.9.0 software (Saeed et al., 2003). Every experiment was carried out in triplicate. Statistical significance from different treatments was revealed after one-way analysis of variance (ANOVA) followed by Tukey’s test. Partial Least Square (PLS) models were performed using SIMCAP+ version 13.0 (Umetrics AB, Umeå, Sweden) with 14 metabolites as X variables and eight biological activities as Y variables. All variables were mean-centered and unit-variance (UV) scaled prior to PLS. Correlation analysis was performed using Past 3.0 (Øyvind Hammer, Natural History Museum, University of Oslo, Oslo, Norway) using the Pearson parametric correlation test and visualized using Heatmapper (Babicki et al., 2016). Significant thresholds at p < 0.05 with significant differences represented by different letters or p < 0.05, <0.01, and <0.001 were used for all statistical tests and represented by different letters or by *, **, and ***, respectively.

## Results

### Establishment of Callus Culture

The most optimal callus induction frequency was established by testing different hormonal combinations of auxins and cytokinins, either alone or in combination (**Table 2**). Media containing cytokinin in combination with auxin resulted in the highest accumulation of callus biomass. Murashige and Skoog (MS) (1962) media supplemented with 2 mg.L^−1^ BAP and 0.5 mg.L^−1^ NAA gave the maximum growth index (**Table 2**).

**Table 2:** Growth indices of *L. usitatissimum* calli grown *in vitro* on Murashige and Skoog media containing different 1-naphthaleneacetic acid (NAA) and 6-benzylaminopurine (BAP) hormonal concentrations after 20 days of culture.

NAA(mg/L)	BAP(mg/L)	Growth index^1^
0.1	1	1.36 ± 0.07^d^
0.5	1	1.43 ± 0.06^d^
1	1	1.76 ± 0.05 ^c^
0.1	2	2.07 ± 0.10^b^
0.5	2	2.79 ± 0.03^a^
1	2	2.67 ± 0.09^a^

^1^Growth index represents the ratio of final biomass (in dry weight (DW)) divided by the initial biomass (in DW) determined at day 20 of cultivation. No callus induction was observed on Murashige and Skoog medium without addition of any phytohormone. Values are means ± SD of 3 independent experiments; superscript letters indicate significant differences (p < 0.05).

Next, we evaluated the influence of the explant origin on callus formation using root, cotyledon, or hypocotyl starting explants. Callus formation was observed for each of these initial explants. Growth index as the ratio between final biomass and initial biomass (*i.e.*, 1 g FW per petri dish for 5 micro-callus) has been deduced for each case for determination at day 20 of cultivation on MS media containing 2 mg.L^−1^ BAP and 0.5 mg.L^−1^ NAA. The present results showed that hypocotyls constituted the best initial explants for the establishment of calli in terms of biomass accumulation (**Table 3**).

**Table 3:** Growth indices in *L. usitatissimum* calli as a function of the initial explant grown on Murashige and Skoog media containing 2 mg.L^−1^ 6-benzylaminopurine (BAP) and 0.5 mg.L^−1^ 1-naphthaleneacetic acid (NAA).

Initial explant origin	Growth index^1^
Root	2.55 ± 0.06^b^
Hypocotyl	2.79 ± 0.03^a^
Cotyledon	2.24 ± 0.07^c^

^1^Growth index represents the ratio of final biomass [in dry weight (DW)] divided by the initial biomass (in DW) determined at day 20 of cultivation. Values are means ± SD of three independent experiments; superscript letters indicate significant differences (p < 0.05).

### Study of Growth Kinetics

**Figure 1:**
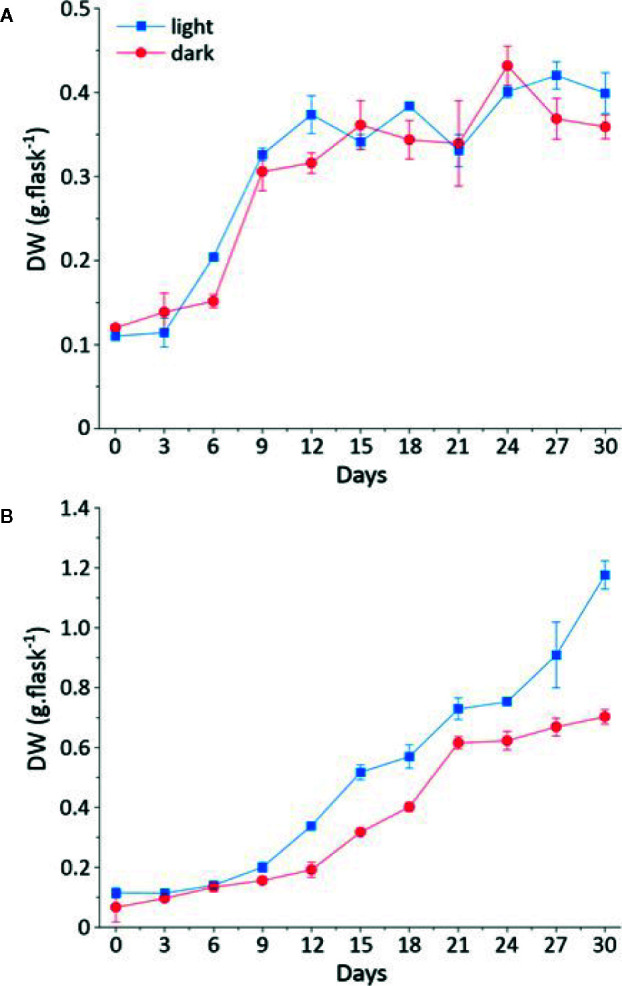
Growth kinetics of callus and cell suspension cultures in light and dark represented in dry weight (in g/flask) on a time period of 30 days. **(A)** Dry weight (DW) of callus grown in the light (Blue); dry weight of callus grown in the dark (Red) **(B)** Dry weight (DW) of cell suspension culture grown in the light (Blue); dry weight of cell suspension culture grown in the dark (Red).

**Figure 2:**
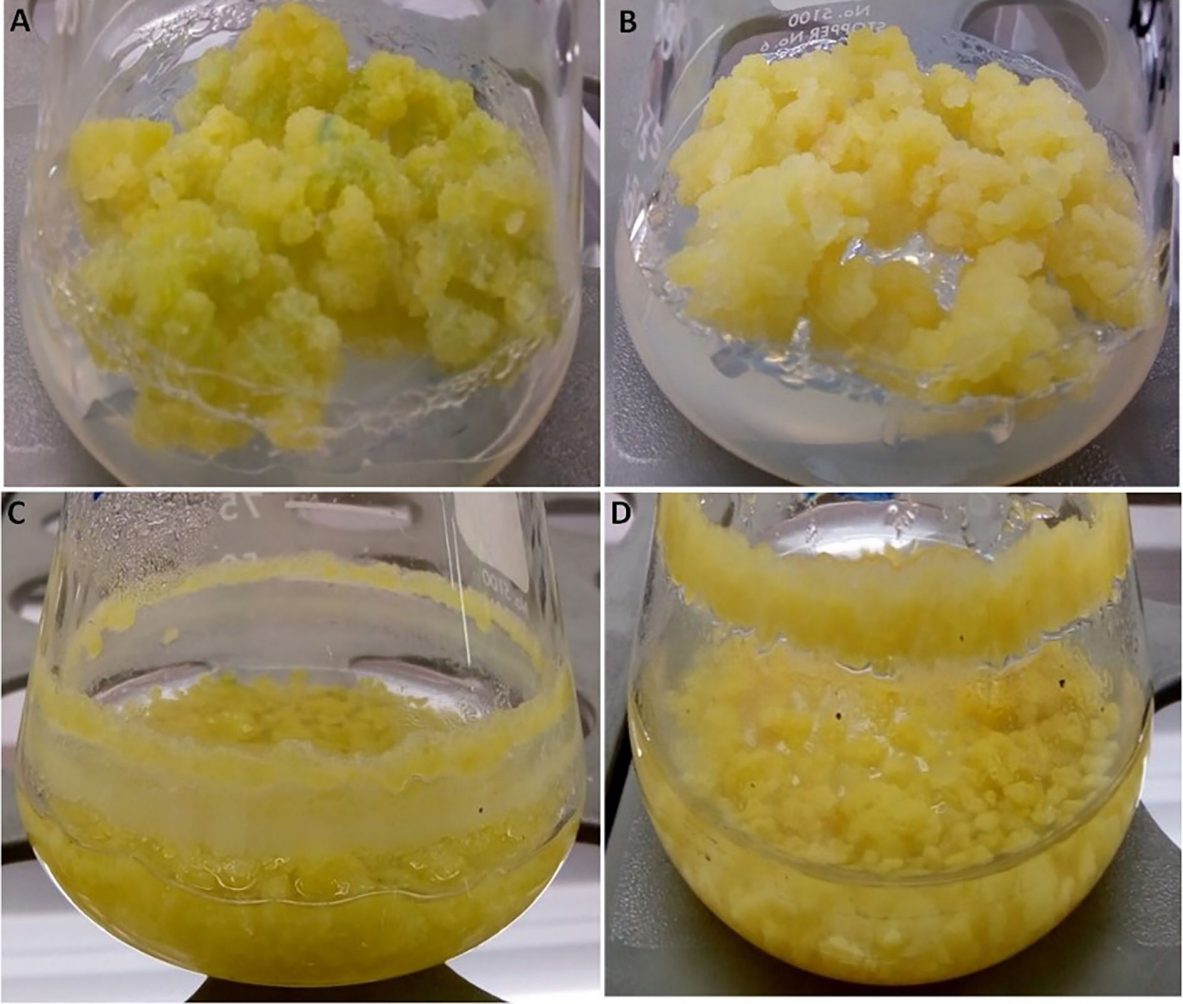
Pictures of representative *L. usitatissimum* cultures. **(A)** Callus culture in the light after 30 days. **(B)** Callus culture in the dark after 30 days. **(C)** Suspension culture in the light after 27 days of culture. Suspension culture in the dark after 24 days of culture **(D)**.

The growth kinetics of cell suspension (**Figure 1A**) and callus (**Figure 1B**) under light or dark were studied in *L. usitatissimum* over a period of 30 days. We decided to focus on dry weight (DW) measurements, as fresh weight (FW) cannot provide an accurate evaluation of biomass production (Park and Kim, 1993). In flax callus cultures the maximum biomass accumulation (DW) was observed on day 30 of culture (**Figure 1B**). In light grown cultures (**Figure 2A**), 1.176 g DW/flask was recorded, while 0.702 g DW/flask was measured for cultures growing in the dark (**Figure 2B**). In both conditions *i.e.* dark and light, the exponential-growth phase started from day 9 of culture, after an initial lag phase. Whereas in cell suspension cultures, the exponential-growth phase was activated earlier on day 3 in the light condition and on day 6 in the dark condition (**Figure 1A**). The highest suspension cell culture DW in the light was recorded on day 27 (0.399 g/flask) whereas in the dark the highest biomass was recorded on day 24 (0.359 g/flask). These two opposing conditions, *i.e.* light (**Figure 2C**) and dark (**Figure 2D**), were studied to see if there was any effect of the photoperiod on biomass accumulation. Light did not have an effect on the growth of suspension cell cultures.

### Identification of Metabolites by UPLC-DAD-MS

To identify the phenolic compounds in cell extracts of *L.* *usitatissimum*, qualitative UPLC-DAD-MS/MS analysis in both ES^+^ and ES^−^ modes were carried out, and major peaks were annotated according to their MS and UV features (**Table 1**, **Figure S1**, and **Table S1**). The 14 major analytes were assigned by comparison with pure standards or data from the literature. Peaks 3, 8, 9, and 11 were undoubtedly identified as *p*-coumaric acid, secoisolariciresinol, lariciresinol, and pinoresinol respectively, by comparison with pure standards. Peaks 1 and 2 show similar MS and UV spectra. In ES^+^ mode the following ions are produced; [M+H-H_2_O]^+^ at *m/z* 521, [M+H-glucose]^+^at *m/z* 377.0, and [M+H-glucose-2H_2_O]^+^ at *m/z* 341.1. In ES^−^ mode, an [M+FA-H]^−^ ion at *m/z* 583.2 and an [M-H-glucose]^−^ ion at *m/z* 375.2 were detected. These spectral features corresponded to the two isomers previously reported in flax cell extracts (Beejmohun et al., 2007) as *erythro* (peak 1) and *threo* (peak 2) forms of the guaiacylglycerol-*β*-coniferyl alcohol ether glucosides (GGCG). Peaks 4 and 7 showed similar MS and UV spectra. In ES^+^ mode they produce the following ions: [M+H-H_2_O]^+^ at *m/z* 503, [M+H-H_2_O-glucose]^+^ at *m/z* 341. In ES^−^ mode, [M+FA-H]^−^ ions at *m/z* 565, [M-H-H_2_O-glucose]^−^ ions at *m/z* 339.1 and [2M-H]^−^ ions at *m/z* 1039 were detected. These chemical features corresponded to dehydrodiconiferyl alcohol-4-*β*-D-glucosides in agreement with Beejmohun et al. (2007). Our analyses enabled the detection of two isomers provisionally assigned as dehydrodiconiferyl alcohol-4-*β*-D-glucoside (DCG) isomer 1 (peak 4) and isomer 2 (peak 7), whereas previous studies reported only one isomer (Attoumbre et al., 2006a; Beejmohun et al., 2007). Peaks 5 and 6 produced similar MS and UV spectra. In ES^+^ mode they produced [M+H-2H_2_O]^+^ ions at *m/z* 341.0, [M+Na]^+^ ions at *m/z* 398.9, and [2M-2H_2_O+H]^+^ ions at *m/z* 717.1. In ES^−^ mode, [M-H]^−^ ions at *m/z* 375, [2M-H]^−^ ions at *m/z* 751.5, [M-H-H_2_O-CH_2_O]^−^ ions at *m/z* 327.1, and [M-H-H_2_O-CH_2_O-CH_3_]^−^ ions at *m/z* 312. 3 were detected. The structures of these two isomers were tentatively identified as *erythro* (peak 5) and *threo* (peak 6) forms of the guaiacylglycerol-*β*-coniferyl alcohol ether (GGC), whereas only their glucoside forms have been previously reported in flax cell extracts (Beejmohun et al., 2007). In ES^+^ mode, peak 10 produced the following ions: [M+H-H_2_O]^+^ at *m/*z 341.1, [M+H-2H_2_O]^+^ at *m/*z 323.0, and [M+H-H_2_O-2CH_3_]^+^ at *m/*z 311.1. In ES^−^ mode an [2M-H]^−^ ion at *m/*z 715.2, [M+FA-H]^−^ ion at *m/*z 403.1, [M-H-H_2_O]^−^ ion at *m/*z 339.1 and [M-H-2CH_3_]^−^ ion at *m/*z 327.2 were detected. These chemical features were similar to those observed for pinoresinol (peak 11) and might be assigned to its enantiomer epipinoresinol. The compound has been previously reported in *Forsythia intermedia* cell suspension cultures (Schmitt and Petersen, 2002) and now, for the first time, in flax cells. In ES^+^ mode, peak 12 produces an [M+H-H_2_O]^+^ ion at *m/*z 339.1, and an [M+H-2H_2_O]^+^ ion at *m/*z 321.0. In ES^−^ mode, the same peak produces an [M-H-H_2_O]^−^ ion at *m/*z 337.1 and an [M+FA-H]^−^ ion at *m/*z 400.9. The formation of the two characteristic fragments [A]^+^ ion at *m/*z 137 and [B]^+^ ion at *m/*z 161 allowed for unambiguously assigning these compounds as pluviatolide as previously described from aerial parts of *L. usitatissimum* (Schmidt et al., 2008). The presence of pluviatolide in *L. usitatissimum* cell suspension cultures is described for the first time in this study. In ES^+^ mode, peak 13 produces an [M+H-H_2_O]^+^ ion at *m/*z 519.2, an [M+H-H_2_O-hexoside]^+^ ion at *m/*z 357.0 (hexose neutral loss: −162 Da). In ES^−^ mode, peak 13 produces an [M-H+FA]^−^ ion at *m/*z 581.1, an [M-H-H_2_O]^−^ ion at *m/*z 517 and an [M-H-H_2_O-hexose]^−^ ion at *m/*z 355 (hexose neutral loss: −162 Da), with the UV spectrum showing a *λ*max at 277 and 344 nm. These MS and UV characteristics corresponded to the hexoside form of guaiacylglycerol-*β*-coniferyl aldehyde ether described by Yao et al. (2018). Consequently, this compound was tentatively assigned as guaiacylglycerol-*β*-coniferyl aldehyde ether hexoside. To our knowledge, we are the first to report its presence in flax cells. In ES^+^ mode peak 14 produces an[M+Na]^+^ ion at *m/*z 394.9, an [M+H-H_2_O-CH_3_]^+^ ion at *m/*z 341.0, and in ES^−^ mode an [M-H-H_2_O]^−^ ion at *m/*z 352.9 and an [M-H-2CH_3_]^−^ ion at *m/*z 341. These MS spectral features enabled us to provisionally assign this compound as phillygenin, as previously described in sesame seed extracts (Eklund et al., 2008).

### Accumulation of Specialized Metabolites

**Figure 3:**
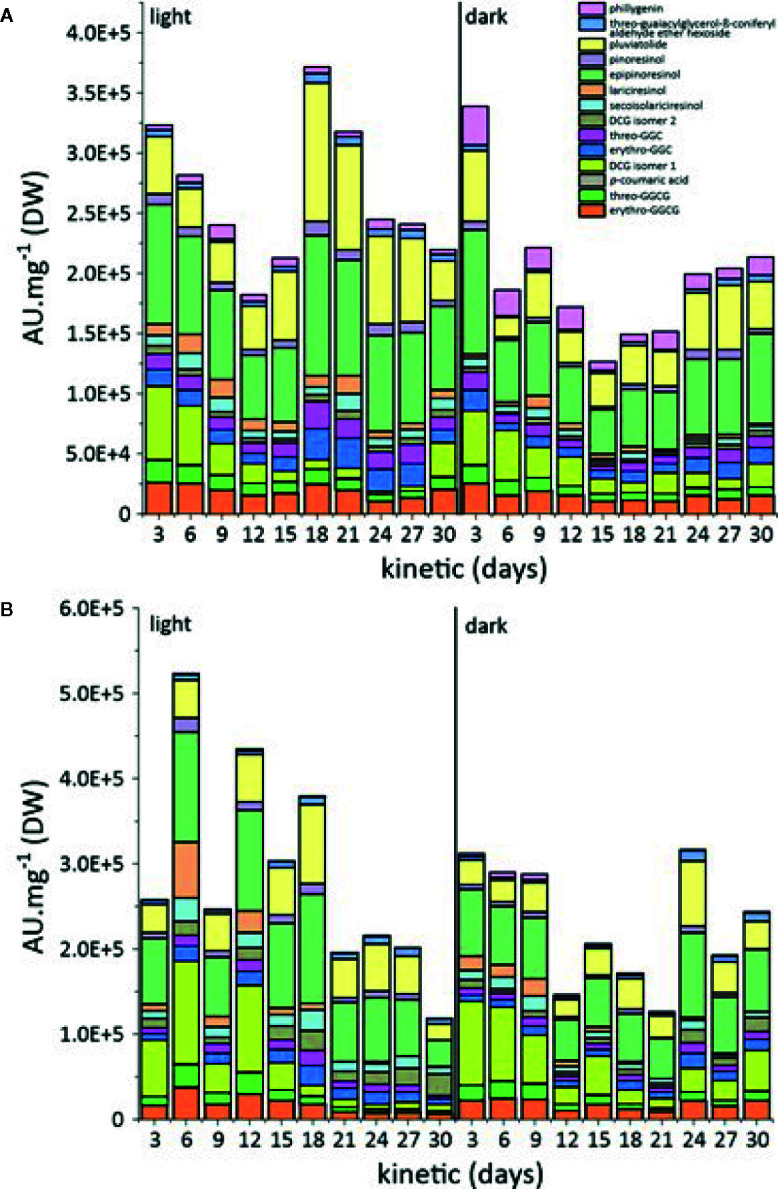
**(A)** Comparison of metabolic variations in *L. usitatissimum* callus cultures in the light and dark. **(B)** Comparison of metabolic variations in *L. usitatissimum* cell suspension cultures in the light and dark. A.U. mg^−1^ refers to the sum of arbitrary unit of each compound per mg of DW.

The accumulation of these compounds in the cell cultures was measured over a period of 30 days. We observed the accumulation of 14 different specialized metabolites in *L. usitatissimum* callus and cell suspension cultures, following UPLC-HRMS analysis (**Figures 3**, **S2**).

The analysis of specialized metabolite accumulation in callus grown under light *versus* dark conditions showed a higher accumulation of these metabolites on day 18 of cultivation under light conditions (**Figures 3A**, **S2A**). Similar results were obtained for cell suspension cultures propagated under light conditions, where the production of specialized metabolites was comparatively higher than under dark conditions (**Figures 3B**, **S2B**).

Epipinoresinol and pluviatolide were the major lignans of flax callus and cell suspension cultures. Their biosynthesis appeared to be growth associated with maximum accumulation at the end of the exponential growth phase. No significant difference was observed between the accumulations in callus *vs.* cell suspension. However, a stimulation of the light on their accumulation has been observed. For the accumulation of other lignans, phillygenin was higher in the dark-grown callus, while secoisolariciresinol and lariciresinol contents were higher in the cell suspension under light conditions. Under these conditions, pinoresinol accumulation did not show any marked variation.

DCG (isomer 1) was the main neolignan produced in both culture and cell suspension conditions. The DCG (isomer 1) accumulation was twofold more important in cell suspension (day 6) than in callus (day 3), with an observed stimulating effect of light. In both types of *in vitro* culture, its maximum accumulation was observed both at the beginning and at the end of the culture cycle. With the exception of *erythro*-guaiacylglycerol-*β*-coniferyl alcohol ether glucoside, *erythro*-guaiacylglycerol-*β*-coniferyl alcohol ether, and *threo*-guaiacylglycerol-*β*-coniferyl alcohol ether, all of which peaked at day 18 in flax cell suspension under light conditions, the accumulation of other neolignans under other conditions remained relatively stable throughout the culture cycle.

### Antioxidant and Anti-Aging Activities

A complete evaluation of antioxidant and anti-aging activities of all the cell extracts of *L. usitatissimum* was performed. In total, four different types of assays were performed in order to provide a complete view on the antioxidant capacities of the extracts: two assays (DPPH and ABTS) to detect antioxidants acting through a hydrogen-atom transfer (HAT) mechanism, and two other assays (CUPRAC and FRAP) acting through a single electron transfer (ET) mechanism (Prior et al., 1998 and Apak et al., 2007).

**Figure 4:**
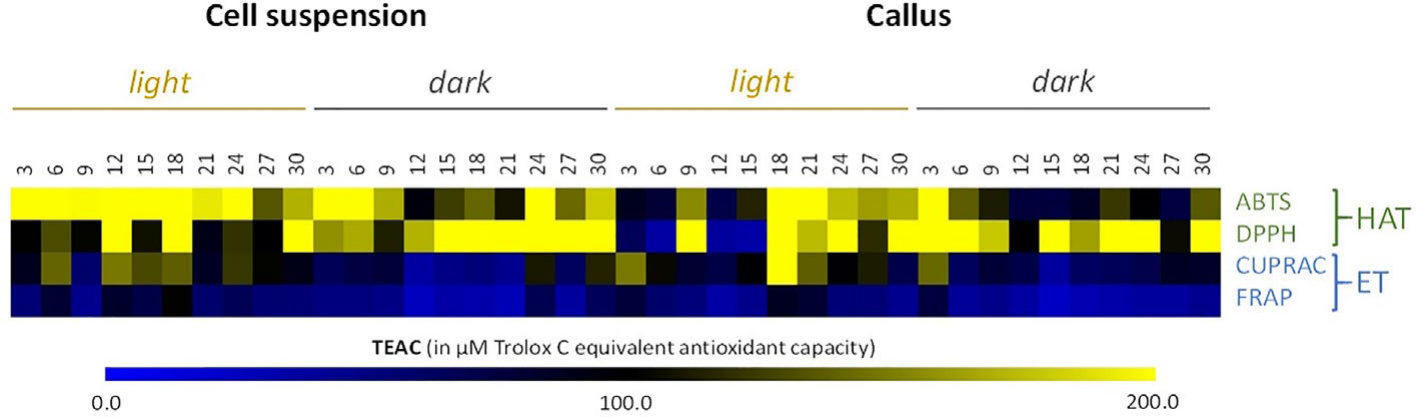
Heat map showing *in vitro* antioxidant activity in all the cell cultures of cell suspensions and callus extracts of *L. usitatissimum* over a time period of 30 days. Antioxidant activities are expressed in µM of Trolox C Equivalent Antioxidant Capacity (TEAC). Values are presented in **Table S2** . DPPH, 1,1-Diphenyl-2-picryl-hydrazyl; ABTS, 2,2-azinobis-(3-ethyl-benzothiazoline-6-sulfonic acid); FRAP, ferric reducing antioxidant power; CUPRAC, cupric reducing antioxidant capacity; HAT, hydrogen atom transfer antioxidant mechanism; ET, electron transfer antioxidant mechanism.

It is clear from **Figure 4** and **Table S2** that ABTS and DPPH activities were higher in comparison to the CUPRAC and FRAP activities. Nonetheless, cell suspension extracts grown under light conditions showed the best result, with the highest antioxidant activity observed on day 18 of culture for all *in vitro* assays (with TEAC of 558.5 µM (ABTS), 334.7 µM (DPPH), 142.8 µM (CUPRAC), and 108.5 µM (FRAP)).

**Figure 5:**
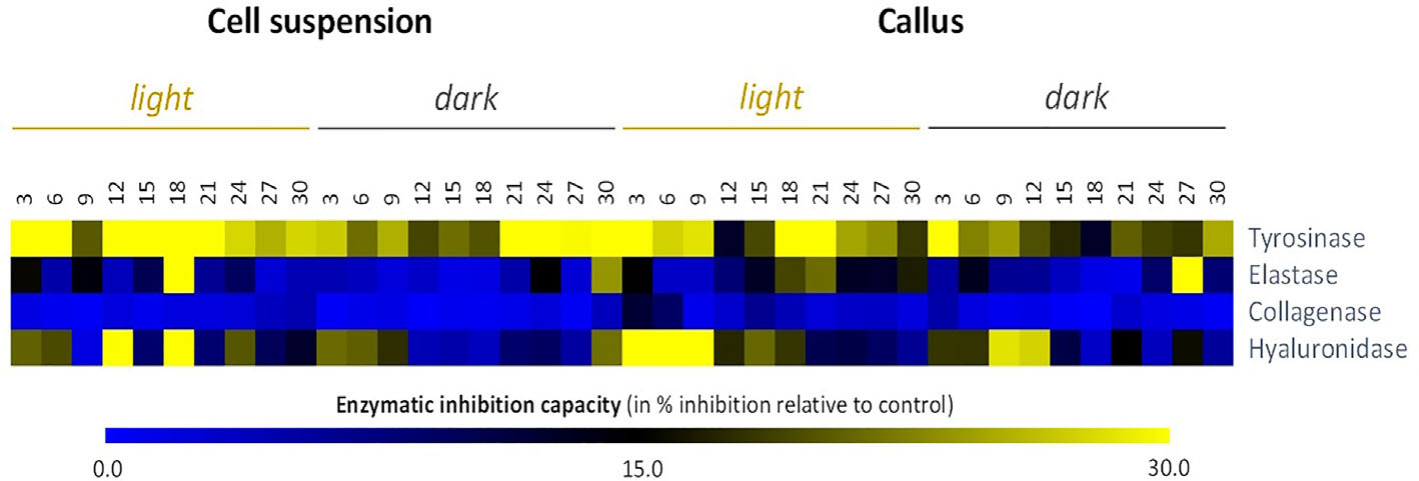
Heat map showing relative *in vitro* anti-aging activity inhibitory activity against skin remodeling enzymes) of cell suspensions and callus extracts of *L. usitatissimum* over a time period of 30 days. *In vitro* anti-aging activities are expressed in inhibition % relative to the control (same volume of extraction solvent). Values are presented in **Table S3**.

Next, we examined the anti-aging capacity of all *L. usitatissimum* cell extracts by performing four different assays. Tyrosinase, elastase, collagenase, and hyaluronidase inhibitors are of great interest to the cosmetics industry. From heat maps (**Figure 5**), it can be interpreted that anti-tyrosinase and anti-elastase activities displayed a comparatively higher propensity in the dark than in the light. But in more details, cell suspension, in particular SL18 extract (*i.e.* cell suspension grown under light conditions on day 18 of culture) presented the maximum inhibitory action for tyrosinase and elastase enzymes with observed inhibitions of 50.6 and 34.9%, respectively (**Figure 5**, **Table S3**). Better results have been achieved with collagenase and hyaluronidase inhibition of callus cultures. Nevertheless, *L. usitatissimum* extracts did not have major inhibitory effects on collagenase enzyme activity, with a maximum inhibition of 11.9% observed for CL3 extract (*i.e.*, callus grown under light conditions on day 3 of culture). On the contrary, the same CL3 extract showed the maximum inhibitory potential for the hyaluronidase enzyme with a 52.8% inhibition observed.

### Multivariate Statistical Analyses

**Figure 6:**
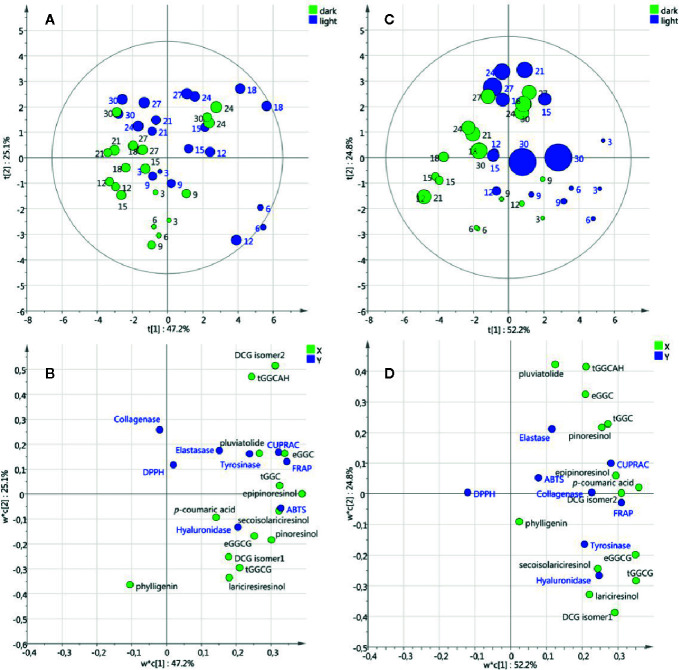
Partial Least Square models of metabolic composition and biological activities of cell suspension **(A, B)** and callus **(C, D)** cultures under light/dark treatment. Score plots **(A, C)** with round size relative to the biomass expressed as dry weight and numbered with the corresponding days of culture. Loading plots **(B, D)** with X variables in green and Y variables in blue.

Partial Least Square models were performed on the data sets from cell suspensions and callus cultures to extract relevant changes of metabolic composition and biological activities under light or dark treatments, as well as over time. For cell suspension cultures, the PLS score plot of the two first components shown in **Figure 6A** explained 72.3% of the variation and revealed a slight effect of the light/dark treatment along component 1 axis. The loading plot (**Figure 6B**) showed the variables potentially responsible for the discriminations observed in **Figure 6A**.

As an example, the projection on component 1 positive values of the neolignans (*erythro*-guaiacylglycerol-*β*-coniferyl alcohol ether, *threo*-guaiacylglycerol-*β*-coniferyl alcohol ether, dehydrodiconiferyl alcohol-4-*β*-D-glucoside isomer2, guaiacylglycerol-*β*-coniferyl aldehyde ether hexoside), the lignans (secoisolariciresinol, epipinoresinol, pinoresinol) and the anti-oxidant tests (ABTS, FRAP and CUPRAC) suggested that under light treatment these metabolites are induced resulting in higher antioxidant activities. It is noteworthy that biomass accumulation (DW and FW) was not associated with the variables corresponding to metabolic composition and biological activities. For callus cultures, the PLS score plot of the two first components shown in **Figure 6C** explained 77% of the variation and a slight effect of light/dark treatment was observed similarly to cell suspension cultures. Interestingly, the variables associated with the light treatment were the same in the cell suspension and callus cultures (**Figures 6B, D**) suggesting that in both callus and cell suspension cultures of *L. usitattissimum* light treatment induced the production of several lignans and neolignans.

**Figure 7:**
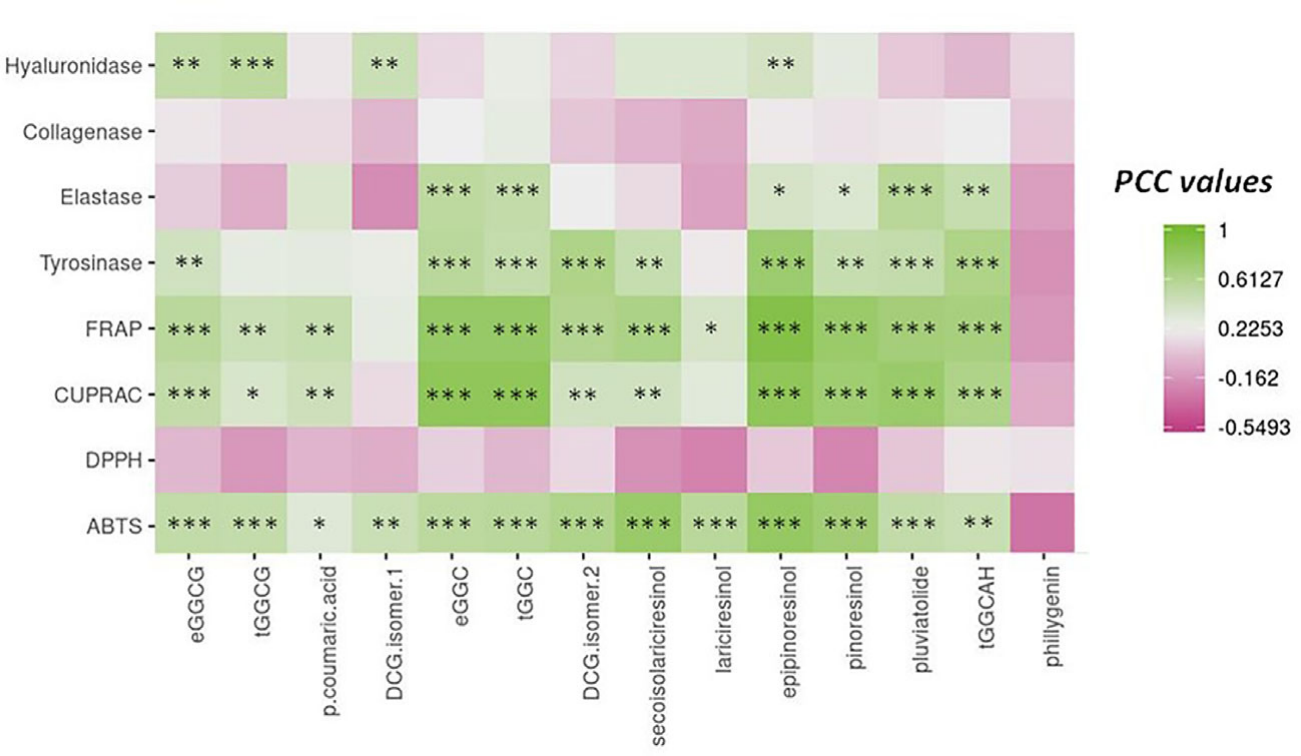
Pearson correlation analysis (PCC) of the relation between the main phytochemicals from flax *in vitro* culture extracts and the different antioxidant (ABTS, DPPH, CUPRAC, and TBARS) and anti-aging (tyrosinase, hyaluronidase, elastase, and collagenase) activities. *** significant p < 0.001; ** significant p < 0.01; * significant p < 0.05; actual PCC values are indicated in **Table S4**.

To evaluate the connection between phytochemicals and biological activities of the extracts, Pearson coefficient correlations (PCCs) were calculated (**Figure 7** **;** **Table S4**). From this analysis, according to their high and significant PCC values, the lignans epipinoresinol, secoisolariciresinol, and pinoresinol and the neolignans dehydrodiconiferyl alcohol-4-*β*-D-glucoside (isomer 2) appeared as the main potential contributors toward the ABTS antioxidant assay. In addition to these compounds, the two neolignans *erythro*- and *threo*-guaiacylglycerol-*β*-coniferyl alcohol ether and guaiacylglycerol-*β*-coniferyl aldehyde ether hexoside were highly correlated to the FRAP antioxidant assay. The same compounds were strongly associated with the CUPRAC antioxidant assay, except for DCG (isomer 2) and secoisolariciresinol. No significant correlation was noted for DPPH radical scavenging assay.

The lignan epipinoresinol and the neolignans dehydrodiconiferyl alcohol-4-*β*-D-glucoside (isomer 2) and guaiacylglycerol-*β*-coniferyl aldehyde ether hexoside emerged as the main possible contributors to tyrosinase enzyme inhibition. A high and significant correlation pointed to the possible implication of pluviatolide in the inhibition of elastase enzyme. A moderate but highly significant correlation between inhibition of hyaluronidase enzyme and neolignans erythro-guaiacylglycerol-*β*-coniferyl alcohol ether glucoside and dehydrodiconiferyl alcohol-4-*β*-D-glucoside (isomer 1) and lignan epipinoresinol was measured.

## Discussion

Flax extract is considered to be a potential cosmetic ingredient all over the world, including China (China Food & Drug Administration, 2015). Therefore, our objective was to characterize cell suspension extracts that could be used within this field. An undoubted advantage of using cell cultures as opposed to whole plants is that they can be used efficiently for a continuous production of bioactive metabolites (Eibl et al., 2018; Georgiev et al., 2018). This in turn guarantees more reproducible production of economically important extracts and under controlled sanitary conditions. Most importantly, the production of these extracts can be adjusted to the demand at any time.

Flax cell suspensions have been proposed as a useful system for the production of plant biomass able to produce and accumulate bioactive compounds (Attoumbre et al., 2006b). *L. usitatissimum* callus-derived cell suspension has been previously initiated from various starting materials: root explants (Attoumbre et al., 2006a; Attoumbre et al., 2006b), hypocotyls (Hano et al., 2006; Corbin et al., 2013a), or shoots (Gabr et al., 2016). Efficiency in obtaining higher biomass was assessed starting with different tissues. Unsurprisingly, considering the high organogenesis competency of hypocotyl epidermal and sub-epidermal cells (Lamblin et al., 2007), these tissues gave the best results. In the present study, the most optimal callus induction frequency was established by testing different hormonal combinations of auxins and cytokinins. Murashige and Skoog media containing cytokinin in combination with auxin (*i.e.*, 2 mg.L^−1^ BAP and 0.5 mg.L^−1^ NAA) resulted in the highest biomass accumulation. Attoumbre et al. (2006b) also reported higher biomass production of *L. usitatissimum* cell suspension in LS medium (Linsmaier and Skoog, 1965) supplemented with NAA at 1 mg.L^−1^, whereas Gabr et al. (2016) reported higher biomass production with B5 medium (Gamborg et al., 1968) supplemented with 2,4D at 1.0 mg.L^−1^ and GA_3_ at 0.5 mg.L^−1^. Here, the optimal hormonal concentrations are in good agreement with our previous results (Hano et al., 2006; Beejmohun et al., 2007; Hano et al., 2008; Corbin et al., 2013a; Corbin et al., 2013b; Markulin et al., 2019). The presence of two lignans (secoisolariciresinol diglucoside [SDG] and lariciresinol diglucoside [LDG]) and two neolignans (dehydrodiconiferyl alcohol glucoside [DCG] and guaiacylglycerol-*β*-coniferyl alcohol ether glucoside [GGCG]) in *in vitro* cultured cells has already been reported in *L. usitatissimum* (Attoumbre et al., 2006a; Attoumbre et al., 2006b; Hano et al., 2006; Hano et al., 2008; Corbin et al., 2013a; Corbin et al., 2013b; Gabr et al., 2016; Anjum et al., 2017a; Anjum et al., 2017b; Zahir et al., 2018; Nadeem et al., 2018; Nadeem et al., 2019; Ahmad et al., 2019; Markulin et al., 2019). In this study, using a high resolving chromatographic method (UPLC), we show for the first time that our newly established flax callus and their corresponding derived cell suspension lines are able to accumulate at least 14 lignans, neolignans, and derivatives. These compounds include the four compounds already described and 10 metabolites never found before in flax cell suspension systems. This shows the versatility of these cell systems and the usefulness of developing new *L usitatissimum* cell lines to enhance specific metabolic development according to targeted applications. Callus and cell suspension of flax of our study presented a similar qualitative metabolic profile. However, stress from the status modification (from callus to the suspension), physiological and/or chronological conditions (*e.g.*, growth) or light conditions may alter the accumulation of each component in the extracts. Light has already been mentioned as a stress inducer in several plant species, which triggers specialized metabolite biosynthesis (Shohael et al., 2006; Younas et al., 2018; Zahir et al., 2018; Shah et al., 2019). In our study, all the 14 annotated metabolites were followed in callus and cell suspensions at light or dark for 30 days in order to estimate the effect of the culture parameters on their accumulation. The sum of lignans and neolignans in both callus and cell suspensions is nearly the same, except for two lignans (epipinoresinol and pluviatolide) and one neolignan (DCG isomer 1). A beneficial influence of light on this accumulation was presumably observed considering their relative higher accumulation. The initial high level of DCG isomer 1 could be due to the osmotic stress during the subculture as it has already been observed with other phenylpropanoids and lignans (Seidel et al., 2002). Interestingly, phillygenin is the only lignan produced in the same amount in callus, whatever the light condition, while the accumulation of phillygenin in the cell suspension was stimulated in the dark.

In a final step, we were interested in the biological activities of interest for cosmetic application by using antioxidant and enzymatic anti-aging tests. We observed that light condition produced cell extracts with higher antioxidant activity, whereas dark condition was linked with a higher anti-aging activity. Therefore, light not only activates biosynthesis of lignans and neolignans in *L. usitatissimum* cells, but also plays a significant role in the biological properties of extracts which have been suggested by Arias et al. (2016) for cell suspension of *Thevenia peruviana*. The correlation analysis of phytochemicals and biological activities contributed to the identification of metabolites correlated with antioxidant or anti-aging activity. Some metabolites are commons for both activity such as epipinoresinol, DCG (isomer 2) and guaiacylglycerol-*β*-coniferyl aldehyde ether hexoside, while others are more directly related to antioxidant activity, such as secoisolariresinol, as it was previously showed for some of its derivatives (Prassad, 1999; Kitts et al., 1999; Hano et al., 2017; Socrier et al., 2019) or anti-aging, such as pluviatolide. Surprisingly, phillygenin was not associated with anti-aging activity, probably due to its narrow accumulation profile, because it was primarily present in dark cultured cells. It is also difficult to conclude that the activity of the extract is attributable to a specific metabolite, although some accumulation patterns of similar metabolites tend to be closely related to certain biological activities. To explore further the properties of each molecule, analysis with purified molecules will be necessary. In fact, the variability in the relative abundance of the extract may also be the key to the possible biological activities of the extract. It is commonly agreed that the biological activities of plant extracts may result in the synergistic action of several metabolites, which can be almost inactive on their own in their purified form. Therefore, the study of a specific combination of such molecules may be interesting. Finally, it is also important to bear in mind that our analysis was centered on lignans and neolignans, but extracts contained several other unidentified compounds that may be part of their biological activity. Nevertheless, our findings reinforced and further strengthened the interest in the cosmetic applications of flax lignans and neolignans produced in plant cell culture grown in *in vitro* systems under distinct light conditions.

## Data Availability Statement

All datasets generated for this study are included in the article/**Supplementary Material**.

## Author Contributions

SB did the research work, data analyses and manuscript write-up. TM, NG-G’H, BA, LG, DT, and BS-P contributed to the experimental design and the analysis of the *in vitro* culture experiments. AL, KB, TM, SM, ED, and MC helped with phytochemical and multivariate statistical analyses. BA and CH have supervised the biological assays. CH, BA, and NG-G'H have contributed to the conception of the project, analyses of results and critically reviewed the manuscript.

## Funding

This research was supported by Cosmetosciences, a global training and research program dedicated to the cosmetic industry. Located in the heart of the Cosmetic Valley, this program led by University of Orleans is funded by the Region Centre-Val de Loire (VALBIOCOSM 17019UNI).

## Conflict of Interest

The authors declare that the research was conducted in the absence of any commercial or financial relationships that could be construed as a potential conflict of interest.

## Supplementary Material

Supplementary Figure 1
**(A)** UPLC-MS chromatogram of 30 days *L. usitatissimum* cell suspension extract using photo-diode array detection. (1) *erythro*-guaiacylglycerol-*β*-coniferyl alcohol ether glucoside, (2) *threo*-guaiacylglycerol-*β*-coniferyl alcohol ether glucoside, (3) *p*-coumaric acid, (4) dehydrodiconiferyl alcohol-4-*β*-D-glucoside isomer 1, (5) *erythro*-guaiacylglycerol-β-coniferyl alcohol ether, (6) *threo*-guaiacylglycerol-*β*-coniferyl alcohol ether, (7) dehydrodiconiferyl alcohol-4-*β*-D-glucoside isomer 2, (8) secoisolariciresinol, (9) lariciresinol, (10) epipinoresinol, (11) pinoresinol, (12) pluviatolide, (13) guaiacylglycerol-*β*-coniferyl aldehyde ether hexoside, (14) phillygenin. Chemical structures of main specialized metabolites accumulated in the callus and suspension cell cultures of *L. usitatissimum*. **(B)** Chemical structures of some of the main specialized metabolites accumulated in the callus and suspension cell cultures of *L. usitatissimum*.

Supplementary Figure 2 Heat map showing relative cell suspensions and callus extracts of *L. usitatissimum* cultivated in the light and dark for 30 days.
