# Supplementary material for: UPLC-HRMS Analysis Revealed the Differential Accumulation of Antioxidant and Anti-Aging Lignans and Neolignans in In Vitro Cultures of Linum usitatissimum L
Source: Front Plant Sci. 2020 Sep 23;11:508658. doi: 10.3389/fpls.2020.508658 (PMC7539065; doi:10.3389/fpls.2020.508658)
Supplement: Supplementary file 1 [file DataSheet_1.pdf]

Supplementary Tables

Table S1: Peak assignment of *L. usitatissimum* cell extracts by UPLC-DAD-MS. Glc: glucose, FA: formic acid; ND: not detected.

Peak	RT (min)	Compound class	Compound assignement	m/z [M+H]$^+$	Major ions ES$^+$	m/z [M-H]$^-$	Major ions ES$^-$	λ_{max} (nm)	References
1	5.66	neolignan	*erythro*-guaiacylglycerol-β-coniferyl alcohol ether glucoside	ND	521.1 [M+H-H2O]$^+$<br>377.0 [M+H-glc]$^+$<br>341.1 [M+H-glc-2H2O]$^+$	ND	583.2 [M+FA-H]$^-$<br>375.2 [M-H-glc]$^-$	215, 260	Beejmohun et al. 2007
2	5.79	neolignan	*threo*-guaiacylglycerol-β-coniferyl alcohol ether glucoside	ND	521.1 [M+H-H2O]$^+$<br>377.0 [M+H-glc]$^+$<br>341.1 [M+H-glc-2H2O]$^+$	ND	583.2 [M+FA-H]$^-$<br>375.2 [M-H-glc]$^-$	215, 259	Beejmohun et al. 2007
3	6.49	phenolic acid	*p*-coumaric acid	ND	ND	163.0	130.9, 103.0	225, 309	standard
4	7.36	neolignan	dehydrodiconiferyl alcohol-4-β-D-glucoside isomer1	ND	503.0 [M+H-H2O]$^+$<br>341.1 [M+H-H2O-glc]$^+$ 323.0, 311.0, 136.9	ND	1039.2 [2M-H]$^-$<br>565.1 [M+FA-H]$^-$<br>339.1 [M-H-H2O-glc]$^-$	221, 276	Beejmohun et al. 2007
5	7.69	neolignan	*erythro*-guaiacylglycerol-β-coniferyl alcohol ether	ND	341.0 [M+H-2H2O]$^+$<br>398.9 [M+Na]$^+$<br>717.1 [2M-2H2O+H]$^+$	375.0	751.5 [2M-H]$^-$<br>327.1 [M-H-H2O-CH2O]$^-$<br>312. 3 [M-H-H2O-CH2O-CH3]$^-$<br>195.1, 165.0	222, 265	Beejmohun et al. 2007
6	7.85	neolignan	*threo*-guaiacylglycerol-β-coniferyl alcohol ether	ND	341.0 [M+H-2H2O]$^+$<br>398.9 [M+Na]$^+$<br>717.1 [2M-2H2O+H]$^+$	375.0	751.5 [2M-H]$^-$<br>327.1 [M-H-H2O-CH2O]$^-$<br>312. 3 [M-H-H2O-CH2O-CH3]$^-$<br>195.1, 165.0	220, 265	Beejmohun et al. 2007
7	8.30	neolignan	dehydrodiconiferyl alcohol-4-β-D-glucoside isomer2	ND	503.0 [M+H-H2O]$^+$<br>341.1 [M+H-H2O-glc]$^+$<br>357.0, 323.0, 311.0	ND	1039.2 [2M-H]$^-$<br>565.1 [M+FA-H]$^-$<br>339.1 [M-H-H2O-glc]$^-$	221, 276	Beejmohun et al. 2007
8	9.42	dibenzylbutane	secoisolariciresinol	ND	345.0 [M+H-H2O]$^+$<br>327.0 [M+H-2H2O]$^+$<br>725.1 [2M+H]$^+$<br>294.8,194.8, 162.9, 137.0	361.2	407.0 [M+FA-H]$^-$<br>300.8, 193.0	280	standard
9	9.77	furan	lariciresinol	ND	721.3 [2M+H]$^+$<br>218.0, 189.0	359.3	405.2 [M+FA-H]$^-$<br>329.1	227, 279	standard
10	9.90	furofuran	epipinoresinol	359.1	341.1 [M+H-H2O]$^+$<br>323.0 [M+H-2H2O]$^+$<br>311.1 [M+H-H2O-2CH3]$^+$<br>279.0 , 250.9, 199.0, 174.9, 160.9, 136.9	357.1	715.2 [2M-H]$^-$<br>403.1 [M+FA-H]$^-$<br>339.1 [M-H-H2O]$^-$<br>327.2 [M-H-2CH3]$^-$	235, 277	Okazawa et al. 2011
11	10.48	furofuran	pinoresinol	359.0	341.0 [M+H-H2O]$^+$<br>323.0 [M+H-2H2O]$^+$<br>311.1 [M+H-H2O-2CH3]$^+$<br>279.1 , 250.9, 199.0, 174.9, 160.9, 136.9	357.1	403.1 [M+FA-H]$^-$<br>339.1[M-H-H2O]$^-$<br>327.2 [M-H-2CH3]$^-$	221, 275	standard
12	11.36	Dibenzylbuty-rolactone	pluviatolide	357.0	339.1 [M+H-H2O]$^+$<br>321.0 [M+H-2H2O]$^+$<br>327.1, 307.0, 161.0, 137.0	355.1	[M-H-H2O]$^-$ 337.1<br>[M+FA-H]$^-$ 400.9<br>325.1, 322.1, 306.6	230, 345	Schmidt et al. 2006

13	11.74	neolignan	guaiacylglycerol-β-coniferyl aldehyde ether hexoside	537.2	519.2 [M+H-H2O]$^+$<br>357.0 [M+H-H2O-glucose]$^+$<br>321.1, 219.1, 137.0, 132.8	534.9	581.1 [M-H+FA]$^-$<br>517.0 [M-H-H2O]$^-$<br>355.0 [M-H-H2O-hexose]$^-$<br>327.1, 325.1, 219.1	276, 345	Woo et al. 2016
14	13.23	furofuran	phillygenin	373.3	394.9 [M+Na]$^+$<br>341.0 [M+H-H2O-CH3]$^+$<br>311.0 279.1, 250.9, 174.9, 161.0, 136.9	371.2	352.9 [M-H-H2O]$^-$<br>340.9 [M-H-2CH3]$^-$<br>327.1, 297.2, 162.7	221, 277	Eklund et al. 2008

Table S2: *In vitro* antioxidant activity of cell suspensions and callus extracts of *L. usitatissimum*

Sample	ABTS	DPPH	CUPRAC	FRAP
SL 3	281.3±28.7 ^{b}	108.3±4.2 ^{d}	94.5±3.3 ^{e}	58.0±3.3 ^{ae}
SL 6	479.0±185.3 ^{ab}	136.1±58.9 ^{bcde}	145.0±10.5 ^{ab}	86.1±13.7 ^{acd}
SL 9	200.4±15.2 ^{bc}	109.8±65.7 ^{cde}	61.4±14.5 ^{efg}	47.6±5.6 ^{ag}
SL 12	449.3±95.3 ^{ab}	228.4±20.5 ^{b}	152.2±7.7 ^{ab}	88.6±5.1 ^{ac}
SL 15	277.3±23.4 ^{b}	113.9±48.9 ^{de}	134.7±3.1 ^{a}	77.4±4.7 ^{acd}
SL 18	558.5±13.8 ^{a}	334.7±136.4 ^{ab}	142.8±0.5 ^{ab}	108.5±4.0 ^{ab}
SL 21	194.2±57.0 ^{bcd}	98.1±21.7 ^{de}	91.5±4.4 ^{e}	62.1±1.5 ^{ae}
SL 24	230.8±44.7 ^{bc}	126.3±46.4 ^{cd}	128.0±28.4 ^{abcd}	71.6±17.0 ^{acd}
SL 27	139.9±22.3 ^{d}	106.5±18.6 ^{d}	109.0±3.6 ^{d}	59.9±2.8 ^{ae}
SL 30	173.4±18.6 ^{cd}	214.3±85.9 ^{abcd}	98.0±0.8 ^{e}	58.9±8.1 ^{ae}
SD 3	268.9±26.2 ^{b}	161.0±54.6 ^{bcd}	70.6±19.1 ^{efg}	54.0±13.6 ^{aeg}
SD 6	228.1±31.2 ^{b}	170.4±86.1 ^{bcd}	78.9±2.4 ^{e}	54.3±1.8 ^{a}
SD 9	171.8±7.7 ^{cd}	119.1±53.1 ^{cde}	82.4±11.6 ^{e}	51.6±4.6 ^{a}
SD 12	101.7±11.6 ^{e}	173.8±0.4 ^{c}	39.2±3.0 ^{f}	27.7±3.2 ^{h}
SD 15	130.4±32.5 ^{de}	289.7±6.2 ^{a}	51.6±1.6 ^{g}	36.7±1.8 ^{g}
SD 18	145.7±25.6 ^{cd}	251.4±31.1 ^{ab}	56.2±7.2 ^{fg}	35.7±2.6 ^{gh}
SD 21	115.1±12.7 ^{de}	322.6±21.2 ^{a}	47.4±2.4 ^{g}	30.7±1.4 ^{h}
SD 24	232.7±16.2 ^{b}	259.0±26.8 ^{ab}	116.8±3.0 ^{c}	72.9±0.8 ^{cd}
SD 27	146.7±0.3 ^{d}	306.5±1.4 ^{a}	67.7±0.1 ^{f}	41.6±2.9 ^{g}
SD 30	182.8±62.1 ^{bcd}	441.8±171.9 ^{a}	120.4±26.2 ^{abcde}	68.7±19.1 ^{ade}
CL 3	94.2±10.0 ^{e}	69.5±10.3 ^{de}	152.7±33.1 ^{abc}	82.8±2.1 ^{ac}
CL 6	84.6±20.8 ^{e}	38.7±3.7 ^{e}	111.0±5.9 ^{cd}	61.9±2.8 ^{ae}
CL 9	158.8±14.2 ^{cd}	217.7±27.0 ^{b}	86.6±12.1 ^{e}	55.9±0.4 ^{af}
CL 12	76.8±2.0 ^{e}	41.1±26.5 ^{e}	77.7±10.5 ^{e}	38.2±1.4 ^{g}
CL 15	121.0±70.5 ^{cde}	36.4±18.1 ^{e}	105.5±28.1 ^{abcde}	48.7±9.6 ^{aefg}
CL 18	326.5±129.3 ^{b}	214.5±13.7 ^{b}	212.3±77.1 ^{ab}	93.2±46.8 ^{abcdef}
CL 21	252.3±116.6 ^{bcd}	176.6±101.3 ^{abcd}	142.7±40.8 ^{abcd}	86.6±30.4 ^{abcdef}
CL 24	177.7±30.2 ^{bcde}	286.9±70.5 ^{ab}	104.9±14.4 ^{cd}	57.5±11.0 ^{aef}
CL 27	165.8±64.9 ^{c}	123.9±25.6 ^{d}	117.9±44.3 ^{abcd}	58.4±21.2 ^{adefg}
CL 30	171.7±8.3 ^{bcd}	246.5±1.9 ^{b}	76.2±1.9 ^{e}	46.9±2.2 ^{a}
CD 3	210.3±64.4 ^{bcde}	330.6±221.6 ^{abcd}	146.5±25.1 ^{ab}	80.7±15.7 ^{ag}
CD 6	142.2±18.3 ^{d}	280.9±19.1 ^{a}	68.1±10.8 ^{ef}	42.7±3.6 ^{ag}
CD 9	118.3±32.0 ^{de}	181.8±128.2 ^{abcde}	83.1±16.8 ^{de}	50.0±6.7 ^{aef}
CD 12	82.8±10.4 ^{e}	106.3±55.9 ^{cde}	76.5±42.5 ^{cdefg}	39.8±17.5 ^{aefg}
CD 15	81.7±5.5 ^{e}	211.5±19.6 ^{b}	41.1±0.5 ^{gh}	24.6±0.1 ^{h}
CD 18	90.9±12.2 ^{e}	167.1±83.1 ^{bcd}	66.4±20.7 ^{efg}	32.6±5.3 ^{g}
CD 21	128.4±7.1 ^{d}	201.6±80.3 ^{abcd}	69.5±13.1 ^{ef}	36.5±6.1 ^{g}
CD 24	106.1±32.9 ^{de}	244.4±0.2 ^{b}	75.7±12.8 ^{e}	41.5±4.2 ^{ag}
CD 27	80.1±4.2 ^{e}	111.9±45.8 ^{d}	91.6±14.6 ^{cde}	41.4±5.8 ^{ag}
CD 30	140.5±19.1 ^{d}	349.9±34.6 ^{a}	88.9±22.6 ^{cde}	48.2±7.4 ^{aefg}

In vitro antioxidant activities are expressed in µM of Trolox C Equivalent Antioxidant Capacity (TEAC). Values are means ± SD. Different letters represent significant differences between the various conditions ($p < 0.05$).

Table S3: *In vitro* anti-aging activity (inhibitory activity against skin remodeling enzymes) of cell suspensions and callus extracts of *L. usitatissimum*

Sample	Tyrosinase	Elastase	Collagenase	Hyaluronidase
SL 3	36.3±4.0 ^{bc}	15.5±3.5 ^{a}	1.7±0.8 ^{e}	20.8±16.7 ^{abcd}
SL 6	35.5±9.3 ^{abcd}	5.0±3.1 ^{abc}	0.9±0.4 ^{e}	19.5±0.3 ^{b}
SL 9	20.3±1.4 ^{e}	14.2±4.2 ^{a}	0.5±0.2 ^{f}	1.8±1.8 ^{d}
SL 12	38.0±2.8 ^{bc}	4.2±2.2 ^{abc}	1.9±1.2 ^{e}	54.9±31.7 ^{a}
SL 15	33.2±0.9 ^{c}	10.2±9.5 ^{ab}	1.1±0.3 ^{e}	8.6±4.2 ^{bcd}
SL 18	50.6±5.5 ^{a}	34.9±29.1 ^{a}	1.9±0.5 ^{e}	32.6±16.2 ^{ab}
SL 21	34.5±4.3 ^{bcd}	6.3±3.2 ^{ab}	1.9±1.8 ^{cde}	8.5±3.7 ^{cd}
SL 24	27.7±4.3 ^{cd}	9.4±6.9 ^{ab}	2.3±0.7 ^{e}	20.2±2.2 ^{ab}
SL 27	25.5±0.7 ^{d}	2.4±1.3 ^{bc}	3.9±0.4 ^{c}	9.7±3.0 ^{bc}
SL 30	27.5±8.7 ^{cde}	4.5±2.5 ^{abc}	4.6±1.7 ^{bc}	12.5±1.9 ^{bc}
SD 3	27.0±4.3 ^{d}	4.5±2.6 ^{abc}	0.5±0.1 ^{f}	21.3±2.3 ^{ab}
SD 6	21.5±1.4 ^{de}	3.8±2.4 ^{abc}	1.3±0.8 ^{e}	20.7±0.0 ^{b}
SD 9	25.4±1.1 ^{d}	2.1±1.9 ^{bc}	1.5±1.2 ^{e}	17.8±2.3 ^{b}
SD 12	19.2±0.0 ^{e}	3.3±2.6 ^{abc}	0.2±0.0 ^{f}	4.4±0.1 ^{d}
SD 15	21.6±2.7 ^{de}	1.4±0.4 ^{c}	0.9±0.4 ^{e}	4.9±1.4 ^{cd}
SD 18	19.9±1.4 ^{e}	1.6±0.4 ^{c}	0.9±0.4 ^{e}	3.7±0.1 ^{e}
SD 21	33.4±1.3 ^{c}	5.0±1.1 ^{ab}	1.3±1.2 ^{e}	8.6±1.0 ^{c}
SD 24	37.0±3.4 ^{bc}	15.1±5.0 ^{a}	2.3±0.4 ^{de}	9.3±2.6 ^{c}
SD 27	29.7±1.4 ^{d}	2.5±1.6 ^{bc}	0.6±0.2 ^{ef}	5.5±0.2 ^{d}
SD 30	40.2±15.1 ^{abcd}	23.8±0.3 ^{a}	3.9±1.6 ^{bcd}	21.7±10.1 ^{abc}
CL 3	35.3±5.1 ^{bcd}	15.1±4.4 ^{a}	11.9±3.8 ^{a}	52.8±2.1 ^{a}
CL 6	27.5±3.5 ^{d}	3.3±2.5 ^{bc}	9.1±4.0 ^{ab}	46.0±8.0 ^{a}
CL 9	28.5±1.0 ^{d}	3.3±3.3 ^{abc}	1.2±0.0 ^{e}	40.5±13.6 ^{a}
CL 12	12.6±3.8 ^{f}	8.1±2.8 ^{a}	2.9±1.7 ^{cd}	17.4±4.9 ^{bc}
CL 15	19.3±3.4 ^{def}	12.6±9.3 ^{ab}	6.5±2.8 ^{abc}	21.1±0.3 ^{b}
CL 18	38.1±11.1 ^{abcd}	19.1±15.2 ^{ab}	4.3±3.7 ^{abcd}	18.2±6.1 ^{abc}
CL 21	31.3±12.6 ^{abcd}	21.4±0.8 ^{a}	2.2±1.3 ^{cd}	10.2±4.8 ^{bcd}
CL 24	24.8±3.4 ^{d}	12.9±4.8 ^{a}	3.3±1.3 ^{cd}	10.8±1.1 ^{c}
CL 27	23.6±5.9 ^{de}	12.6±2.0 ^{a}	3.4±0.3 ^{cd}	9.3±1.2 ^{c}
CL 30	18.3±1.6 ^{e}	16.8±16.2 ^{abc}	1.8±1.7 ^{cd}	6.4±0.8 ^{cd}
CD 3	39.5±2.8 ^{b}	5.7±1.2 ^{a}	5.1±1.3 ^{bc}	18.2±3.2 ^{b}
CD 6	22.7±1.8 ^{d}	13.2±8.7 ^{a}	1.9±0.5 ^{e}	18.1±1.7 ^{b}
CD 9	24.3±3.4 ^{d}	6.5±4.4 ^{ab}	1.0±0.1 ^{e}	28.5±5.2 ^{ab}
CD 12	19.7±3.8 ^{def}	6.4±4.9 ^{abc}	1.4±0.8 ^{e}	27.5±13.9 ^{ab}
CD 15	17.4±3.3 ^{ef}	3.6±0.2 ^{b}	0.4±0.0 ^{f}	10.9±0.3 ^{c}
CD 18	18.8±6.2 ^{def}	7.5±4.6 ^{ab}	1.7±1.6 ^{de}	7.3±0.7 ^{c}
CD 21	20.7±2.1 ^{de}	1.0±0.5 ^{c}	3.0±1.3 ^{cd}	14.9±6.8 ^{abc}
CD 24	18.9±0.7 ^{e}	8.9±4.6 ^{a}	1.7±0.4 ^{e}	3.8±0.7 ^{d}
CD 27	18.3±5.7 ^{ef}	30.0±21.3 ^{a}	1.4±1.2 ^{e}	15.8±8.5 ^{abc}
CD 30	25.0±2.4 ^{de}	8.2±1.0 ^{a}	0.6±0.6 ^{de}	5.9±0.3 ^{d}

In vitro anti-aging activities are expressed in inhibition % relative to the control (same volume of extraction solvent). Values are means ± SD. Different letters represent significant differences between the various conditions ($p < 0.05$).

Table S4: Pearson correlation analysis (PCC) of the relation between the main phytochemicals from flax *in vitro* culture extracts and the different antioxidant (ABTS, DPPH, CUPRAC and TBARS) and anti-aging (tyrosinase, hyaluronidase, elastase and collagenase) activities *** significant $p < 0.001$; ** significant $p < 0.01$; * significant $p < 0.05$; NS : not significant at $p < 0.05$.

	e-GGCG	*t*-GGCG	*p*-coum	DCG (iso1)	*e*-GGC	*t*-GGC	DCG (iso2)	seco	lari	epipino	pino	pluviatolide	*t*-GGCAG	phillygenin
ABTS	0.509 ***	0.489 ***	0.327 *	0.451 **	0.547 ***	0.556 ***	0.616 ***	0.768 ***	0.564 ***	0.798 ***	0.707 ***	0.511 ***	0.469 **	NS
DPPH	NS	NS	NS	NS	NS	NS	NS	NS	NS	NS	NS	NS	NS	NS
CUPRAC	0.511 ***	0.375 *	0.440 **	NS	0.819 ***	0.824 ***	0.422 **	0.424 **	NS	0.821 ***	0.735 ***	0.766 ***	0.635 ***	NS
FRAP	0.560 ***	0.446 **	0.476 **	NS	0.782 ***	0.773 ***	0.604 ***	0.658 ***	0.382 *	0.896 ***	0.756 ***	0.705 ***	0.680 ***	NS
Tyrosinase	0.426 **	NS	NS	NS	0.557 ***	0.501 ***	0.634 ***	0.483 **	NS	0.744 ***	0.478 **	0.491 ***	0.635 ***	NS
Elastase	NS	NS	NS	NS	0.549 ***	0.519 ***	NS	NS	NS	0.394 *	0.337 *	0.581 ***	0.482 **	NS
Collagenase	NS	NS	NS	NS	NS	NS	NS	NS	NS	NS	NS	NS	NS	NS
Hyaluronidase	0.517 **	0.549 ***	NS	0.451 **	NS	NS	NS	NS	NS	0.405 **	NS	NS	NS	NS

e-GGCG: erythro-guaiacylglycerol-*β*-coniferyl alcohol ether glucoside; *t*-GGCG: threo-guaiacylglycerol-*β*-coniferyl alcohol ether glucoside; *p*-coum: *p*-coumaric acid; DCG (iso1): dehydrodiconiferyl alcohol-4-*β*-D-glucoside isomer 1; *e*-GGC: erythro-guaiacylglycerol-*β*-coniferyl alcohol ether; *t*-GGC: threo-guaiacylglycerol-*β*-coniferyl alcohol ether; DCG (iso2): dehydrodiconiferyl alcohol-4-*β*-D-glucoside isomer 2; seco: Secoisolariciresinol; lari: Lariciresinol; epipino: Epipinoresinol; pino: Pinoresinol; *t*-GGCAG: guaiacylglycerol-*β*-coniferyl aldehyde ether hexoside.
