# Supplementary figures and images for: UPLC-HRMS Analysis Revealed the Differential Accumulation of Antioxidant and Anti-Aging Lignans and Neolignans in In Vitro Cultures of Linum usitatissimum L
Source: Front Plant Sci. 2020 Sep 23;11:508658. doi: 10.3389/fpls.2020.508658 (PMC7539065; doi:10.3389/fpls.2020.508658)

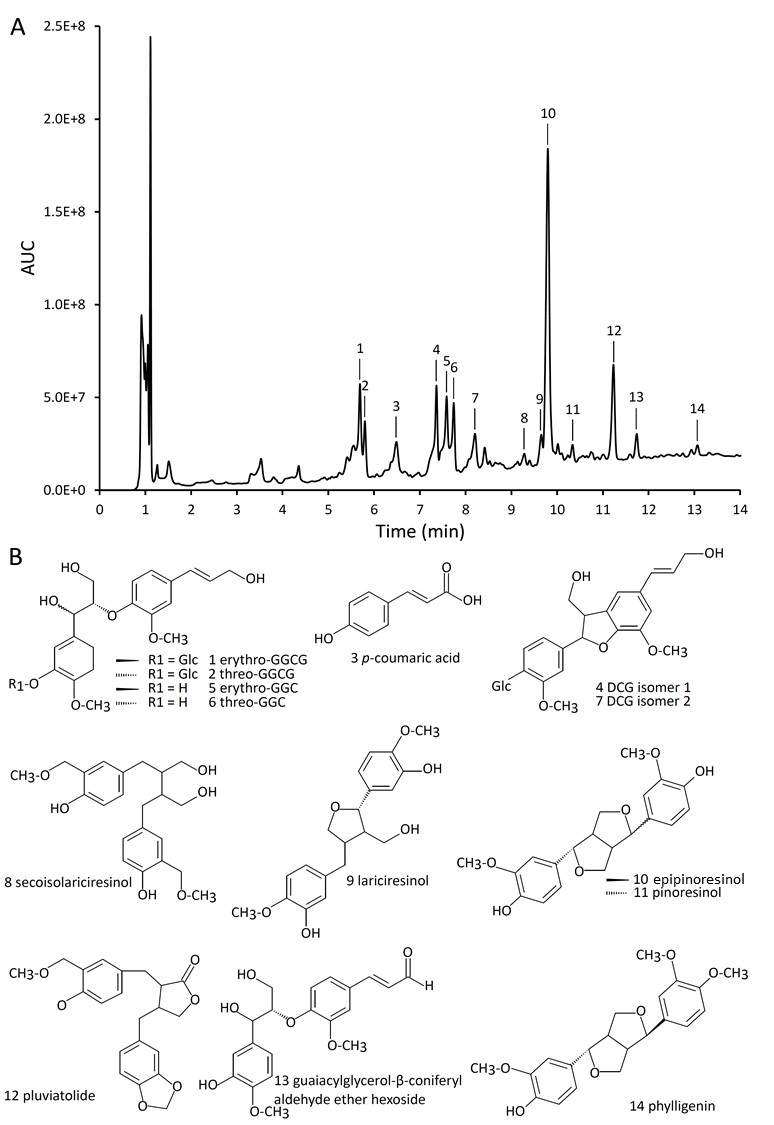

Supplement: Supplementary Figure 1 — (A) UPLC-MS chromatogram of 30 days L. usitatissimum cell suspension extract using photo-diode array detection. (1) erythro-guaiacylglycerol-β-coniferyl alcohol ether glucoside, (2) threo-guaiacylglycerol-β-coniferyl alcohol ether glucoside, (3) p-coumaric acid, (4) dehydrodiconiferyl alcohol-4-β-D-glucoside isomer 1, (5) erythro-guaiacylglycerol-β-coniferyl alcohol ether, (6) threo-guaiacylglycerol-β-coniferyl alcohol ether, (7) dehydrodiconiferyl alcohol-4-β-D-glucoside isomer 2, (8) secoisolariciresinol, (9) lariciresinol, (10) epipinoresinol, (11) pinoresinol, (12) pluviatolide, (13) guaiacylglycerol-β-coniferyl aldehyde ether hexoside, (14) phillygenin. Chemical structures of main specialized metabolites accumulated in the callus and suspension cell cultures of L. usitatissimum. (B) Chemical structures of some of the main specialized metabolites accumulated in the callus and suspension cell cultures of L. usitatissimum. [file Image_1.jpeg]

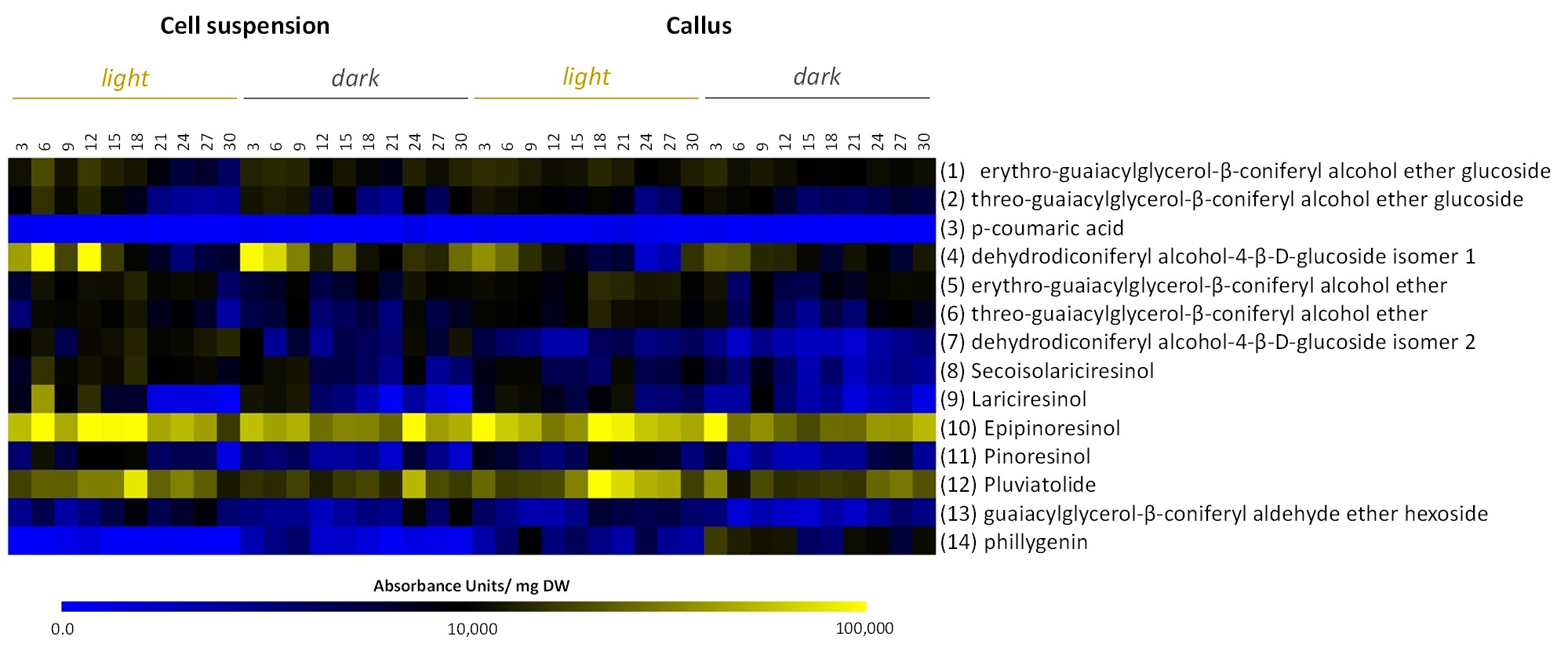

Supplement: Supplementary Figure 2 — Heat map showing relative cell suspensions and callus extracts of L. usitatissimum cultivated in the light and dark for 30 days. [file Image_2.jpeg]
